# Analysis of Boswellic Acid Contents and Related Pharmacological Activities of Frankincense-Based Remedies That Modulate Inflammation

**DOI:** 10.3390/ph14070660

**Published:** 2021-07-10

**Authors:** Friedemann Börner, Markus Werner, Johannes Ertelt, Jürgen Meins, Mona Abdel-Tawab, Oliver Werz

**Affiliations:** 1 Department of Pharmaceutical/Medicinal Chemistry, Institute of Pharmacy, Friedrich-Schiller-University, Philosophenweg 14, 07743 Jena, Germany; friedemann.boerner@uni-jena.de (F.B.); werner.markus@uni-jena.de (M.W.); 2 Heidelberg-Apotheke/AureliaSan GmbH, Steinhofener Str. 12-16, 72406 Bisingen, Germany; johannes@ertelt.de; 3 Central Laboratory of German Pharmacists, Carl-Mannich-Str. 20, 65760 Eschborn, Germany; j.meins@zentrallabor.com (J.M.); tawab@em.uni-frankfurt.de (M.A.-T.); 4 Institute of Pharmaceutical Chemistry, Johann Wolfgang Goethe University, Max-von-Laue-Straße 9, 60438 Frankfurt am Main, Germany

**Keywords:** frankincense, boswellic acids, 5-lipoxygenase, microsomal prostaglandin E_2_ synthase-1, inflammation

## Abstract

Extracts of frankincense, the gum resin of *Boswellia* species, have been extensively used in traditional folk medicine since ancient times and are still of great interest as promising anti-inflammatory remedies in Western countries. Despite their common therapeutic use and the intensive pharmacological research including studies on active ingredients, modes of action, bioavailability, pharmacokinetics, and clinical efficacy, frankincense preparations are available as nutraceuticals but have not yet approved as a drug on the market. A major issue of commercially available frankincense nutraceuticals is the striking differences in their composition and quality, especially related to the content of boswellic acids (BAs) as active ingredients, mainly due to the use of material from divergent *Boswellia* species but also because of different work-up and extraction procedures. Here, we assessed three frequently used frankincense-based preparations for their BA content and the interference with prominent pro-inflammatory actions and targets that have been proposed, that is, 5-lipoxygenase and leukotriene formation in human neutrophils, microsomal prostaglandin E_2_ synthase-1, and inflammatory cytokine secretion in human blood monocytes. Our data reveal striking differences in the pharmacological efficiencies of these preparations in inflammation-related bioassays which obviously correlate with the amounts of BAs they contain. In summary, high-quality frankincense extracts display powerful anti-inflammatory effectiveness against multiple targets which can be traced back to BAs as bioactive ingredients.

## 1. Introduction

Frankincense, the gum resin from various *Boswellia* species, is an ancient remedy used for centuries in Asian and African folk medicine, especially in traditional Ayurvedic medicine in India [1,2,3]. Preparations from frankincense have been externally applied for the treatment of wounds and skin disorders but also internal use has long been commonly practiced in order to cure a variety of inflammatory and infectious diseases [2]. In Western societies and modern folk medicine, frankincense preparations are among the most popular natural remedies for treatment of inflammatory disorders such as rheumatoid arthritis and osteoarthritis, inflammatory bowel diseases, multiple sclerosis, respiratory diseases including asthma, and cancers [4,5,6,7]. The interest in frankincense-based medicine is still rising due to continuous reports about beneficial effects in numerous (pilot) clinical trials where efficacy and safety have been demonstrated [4,7]. Moreover, frankincense typically contains boswellic acids (BAs) that are unique pentacyclic triterpene acids with multiple pharmacological actions (e.g., inhibition of 5-lipoxygenase (5-LOX), I-κB kinases, cathepsin G, LL-37, or microsomal prostaglandin E_2_ synthase (mPGES)-1) seemingly accounting for the anti-inflammatory and antitumoral properties [4,6,8]. 

Given the expanding market of dietary botanicals not undergoing any pre-marketing authorization for quality and efficacy, concerns regarding quality issues are justified. Several studies revealed, aside from economic adulteration, significant differences in the amount of active ingredients compared to the label. In addition, misleading labels are common, although prohibited by regulation [9,10,11]. In consideration of the increasing popularity of frankincense-based remedies and the justified quality problems associated with dietary botanicals, a preliminary survey was recently conducted on the quality of frankincense-containing dietary botanicals [12]. Among other deficiencies, 41% of the 17 tested top-selling products from the USA and Europe did not comply with label declaration. This was verified by another study, demonstrating that the total contents (*w*/*w*) of the six BAs and two lupeolic acids varied between 0.4 to 35.7% in 17 tested frankincense nutraceuticals purchased from USA, Europe, and India [13]. In fact, this study demonstrated a clear correlation between the BA contents and the tested inhibitory effect on the pro-inflammatory cytokines tumor necrosis factor (TNF)-α, interleukin (IL)-1β, IL-6, and IL-8, and the exerted cytotoxic effect against triple-negative breast cancer cell lines could be further proven in vitro. 

In an attempt to combat the jumble of qualitatively different frankincense products in the botanical dietary market, capsules containing *Boswellia serrata* extracts of the highest pharmaceutical quality complying with the requirements of the European Pharmacopoeia monograph for “Indian Frankincense” (monograph 10.0/2310) are now available for therapeutic use. However, the specifications for Indian frankincense to be classified as an extract of pharmaceutical quality are very restricted, not exceeding a demand of at least 1% content of the lead compounds 11-keto-β-BA (KBA) and 3-acetyl-11-keto-β-BA (AKBA). Meanwhile, a plethora of scientific studies in the last years revealed that other BAs (e.g., βBA and AβBA) also display anti-inflammatory effects with even higher potencies than KBA and AKBA for relevant inflammatory molecular targets such as LL-37, cathepsin G, or mPGES-1 [4]. 

The present study aimed at analyzing the comparability of frequently used commercial *Boswellia serrata*-based remedies for pharmaceutical quality, employing (i) “Sallaki^®^ Tablets”, an Indian authorized medicinal product, (ii) “H15 Ayurmedica^®^”, representing a renowned German frankincense-containing dietary botanical, and (iii) “BOSWELLIASAN^®^” that are Indian frankincense capsules. For this purpose, the content of the six major BAs (KBA, AKBA, βBA, AβBA, α-BA, and AαBA) were determined in each preparation, and the efficiency to interfere with inflammation-relevant molecular targets (i.e., 5-LOX, mPGES-1, and cytokines) was evaluated in cell-free and/or cell-based models using appropriate cell types.

## 2. Results

### 2.1. Analytical Assessment of Boswellic Acids in Frankincense-Based Remedies

**Figure 1:**
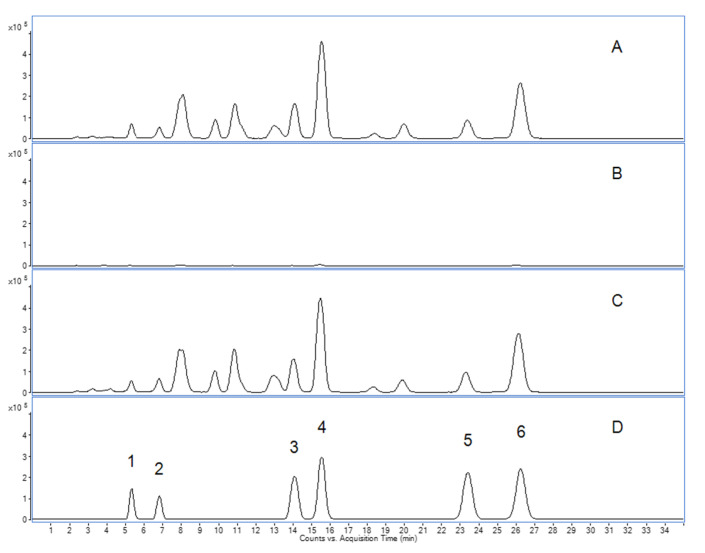
Representative TIC-chromatograms of frankincense-based products. (**A**) “Sallaki^®^ Tablets”. (**B**) “H15 Ayurmedica^®^”. (**C**) “BOSWELLIASAN^®^”. (**D**) Standard solution with 1.2 µg/mL of each BA. 1 = KBA, 2 = AKBA, 3 = α-BA, 4 = β-BA, 5 = Acetyl-α-BA, 6 = Acetyl-β-BA. Experimental conditions and parameter can be found in the Method section.

The quantification of the six characteristic BAs revealed two different fingerprint profiles (Figure 1). Thus, the analytical fingerprints of “Sallaki^®^ Tablets” and “BOSWELLIASAN^®^” were easily distinguishable from that of “H15 Ayurmedica^®^”. Whereas both “Sallaki^®^ Tablets” and “BOSWELLIASAN^®^” contained the six characteristic BAs in plausible amounts, “H15 Ayurmedica^®^” contained only traces of the determined BAs not exceeding 1.92 mg/capsule. Moreover, the amounts of the non-acetylated non-ketylated BAs (αBA, βBA) were found to be higher than the respective acetylated non-ketylated BAs (AαBA, AβBA) in “Sallaki^®^ Tablets” and “BOSWELLIASAN^®^” compared to “H15 Ayurmedica^®^” as can be seen in Table 1.

### 2.2. Inhibition of 5-Lipoxygenase and Microsomal Prostaglandin E_2_ Synthase-1 by Frankincense-Based Remedies

**Figure 2:**
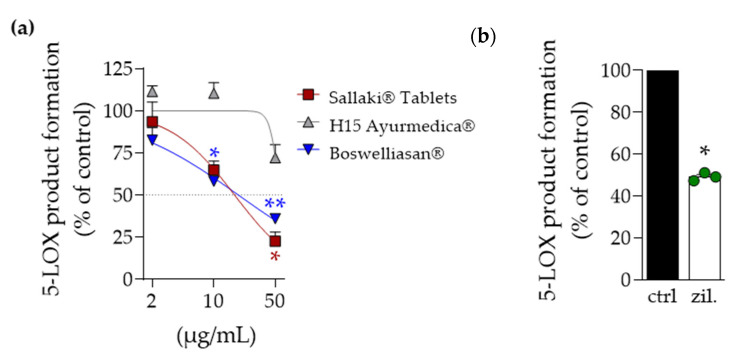
Effects of frankincense-based remedies on 5-LOX activity. (**a**) Purified human recombinant 5-LOX was incubated with 2, 10, or 50 µg/mL of frankincense-based remedies or vehicle (0.1% EtOH) for 15 min at 4 °C and stimulated with 20 µM AA and 2 mM CaCl_2_ at 37 °C. Formed 5-LOX products (sum of tr-LTB_4_, etr-LTB_4_, 5-H(P)ETE) were purified by SPE and analyzed using RP-HPLC. Data, expressed as percentage of control, are given as mean ± S.E.M., n = 3; repeated measures ANOVA + Dunnett post hoc test with raw data; * *p* < 0.05; ** *p* < 0.01; vs. vehicle. (**b**) Zileuton (3 µM) was used as positive control under the same experimental conditions as above. Paired *t*-test; * *p* < 0.05 vs. vehicle.

In order to study the effectiveness of the three frankincense-based remedies for inhibition of 5-LOX and mPGES-1, we performed well-documented cell-free activity assays of the respective human enzymes. Purified human recombinant 5-LOX was pre-incubated with the test items for 15 min at 4 °C and the enzymatic conversion of exogenously added AA (20 µM) the products tr-LTB_4_, etr-LTB_4_, and 5-H(P)ETE were assessed by RP-HPLC; the 5-LOX inhibitor zileuton was used as reference drug. As shown in Figure 2a, “Sallaki^®^ Tablets” and “BOSWELLIASAN^®^” concentration-dependently inhibited 5-LOX product formation with IC_50_ values of 17 and 19 µg/mL, respectively. In contrast, “H15 Ayurmedica^®^” was hardly active and even at the highest concentration tested (50 µg/mL) suppressed 5-LO activity by only ~30% (Figure 2). Zileuton at 3 µM inhibited 5-LOX activity by 50% (Figure 2b), as expected [14]. 

**Figure 3:**
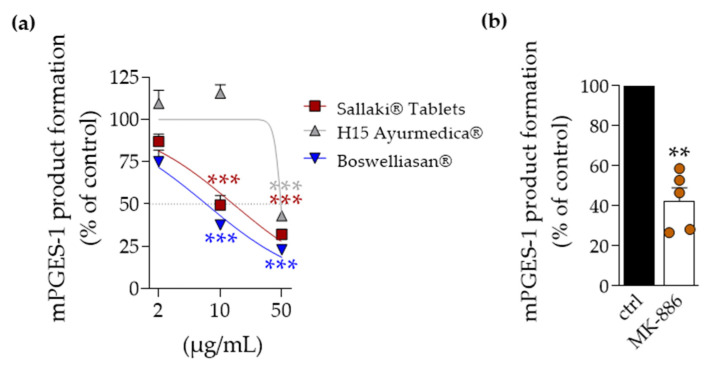
Effects of frankincense-based remedies on mPGES-1 activity. (**a**) Microsomal preparations of IL-1β-stimulated A549 cells were incubated with 2, 10, or 50 µg/mL of frankincense-based remedies or vehicle (0.1% EtOH) for 15 min at 4 °C. The reaction was started with 20 µM PGH_2_. After 1 min at 4 °C, the reaction was terminated using a stop solution containing FeCl_2_ and 11β-PGE_2_. Formed PGE_2_ was purified by SPE and analyzed using RP-HPLC. Data, expressed as percentage of control, are given as mean ± S.E.M., n=5; repeated measures ANOVA + Dunnett post hoc test with raw data; ** *p* < 0.01; *** *p* < 0.001 vs. vehicle. (**b**) MK-886 (10 µM) was used as positive control under the same experimental conditions as above. Paired *t*-test; ** *p* < 0.01 vs. vehicle.

The activity of mPGES-1 was assayed in microsomes of IL-1β-activated human A549 cells that received 20 µM PGH_2_ as substrate for enzymatic conversion to PGE_2_. Pre-treatment with “BOSWELLIASAN^®^” caused most potent suppression of mPGES-1 activity (IC_50_ = 6.9 µg/mL) followed by “Sallaki^®^ Tablets” (IC_50_ = 14 µg/mL) while “H15 Ayurmedica^®^” was again less active (IC_50_ approx. 50 µg/mL) (Figure 3a). MK-886, used as reference inhibitor, suppressed mPGES-1 activity (Figure 3b), as expected [14].

### 2.3. Modulation of Lipid Mediator Production in Exotoxin-Stimulated Neutrophils by Frankincense-Based Remedies

**Figure 4:**
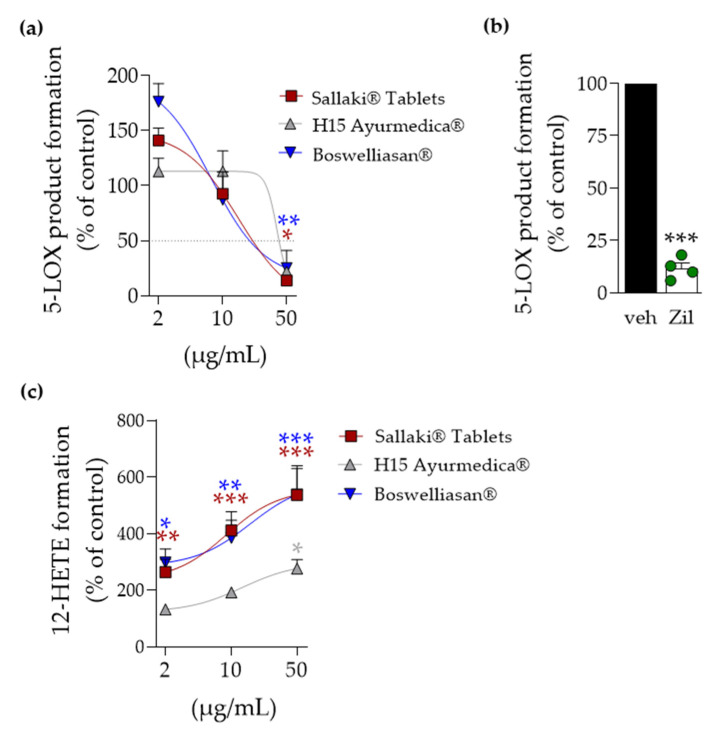
Modulation of LOX product formation in exotoxin-stimulated neutrophils by frankincense-based remedies. Human neutrophils (5 × 10^6^ cells/mL) were incubated with 2, 10, or 50 µg/mL of frankincense-based remedies, 3 µM zileuton, or vehicle (0.1% EtOH) for 15 min at 37 °C. LOX product formation was induced by addition of SACM (1%) and CaCl_2_ (1 mM) for 90 min at 37 °C. Formed lipid mediators (LM) were isolated by SPE and analyzed using UPLC-MS-MS. Data are means ± S.E.M, n = 4–5 separate donors. (**a**) Inhibition of 5-LOX product formation (sum of tr-LTB_4_, etr-LTB_4_, LTB_4_, 5-HETE) by frankincense-based remedies. (**b**) Inhibition of 5-LOX product formation by zileuton (3 µM) under the same conditions. (**c**) Stimulation of 12-LOX product formation (12-HETE) by frankincense-based remedies under the same conditions. Data, expressed as percentage of vehicle control (= 100%), are given as mean ± S.E.M., * *p* < 0.05; ** *p* < 0.01; *** *p* < 0.001 vs. vehicle. ANOVA + Dunnett post hoc test with logarithmized raw data; zileuton (3 µM) was used as positive control under the same experimental conditions as above. T-test; *** *p* < 0.001 vs. vehicle; outliers were identified by using Grubb’s outlier test, α = 0.05.

Next, we assessed how the frankincense-based preparations affect 5-LOX activity in intact human neutrophils that are major producers of leukotrienes [15], challenged with *Staphylococcus aureus*-conditioned medium (SACM) containing exotoxins for eliciting 5-LOX product formation [16]. Freshly isolated neutrophils from human blood were pre-incubated with the frankincense-based remedies for 15 min prior to addition of 1% SACM for 90 min at 37 °C. Formed 5-LOX products (LTB_4_ and its trans-isomer and 5-HETE) were assessed by UPLC-MS-MS as reported [16]. In agreement with the results using isolated 5-LOX, “Sallaki^®^ Tablets” and “BOSWELLIASAN^®^” caused potent suppression of 5-LOX product formation with IC_50_ of 15 and 7.5 µg/mL, respectively, while “H15 Ayurmedica^®^” was less efficient (IC_50_ = 39 µg/mL) (Figure 4a). Notably, the well-known 5-LOX-stimulatory effects in neutrophils at low frankincense extract concentration [17] were evident for “Sallaki^®^ Tablets” and “BOSWELLIASAN^®^” (at 2 µg/mL) but not for “H15 Ayurmedica^®^” (Figure 4a); zileuton at 3 µM markedly suppressed 5-LOX product formation as well (Figure 4b). Furthermore, 12-HETE formation was concentration-dependently elevated by “Sallaki^®^ Tablets” and “BOSWELLIASAN^®^”, as expected based on an AKBA-mediated regiospecificity shift of 5-LOX towards a 12-lipoxygenating enzyme [18], but much less by “H15 Ayurmedica^®^” (Figure 4c) where AKBA contents are minute (see Table 1).

### 2.4. Effects of Frankincense-Based Remedies on Pro-Inflammatory Cytokine Secretion

**Figure 5:**
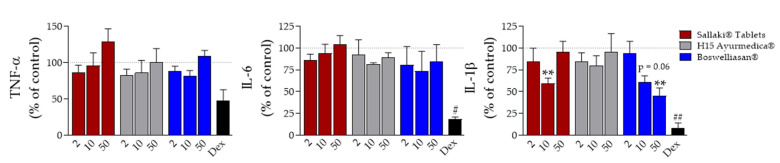
Effects of frankincense-based remedies on cytokine production. Freshly isolated human monocytes (1.5 × 10^6^/mL) were pre-incubated with 2, 10, or 50 µg/mL of frankincense-based remedies, 100 nM dexamethasone, or vehicle (0.1% EtOH) for 30 min. Cells were then stimulated with LPS (10 ng/mL) for 4 h (TNF-α) or 18 h (IL-6, IL-1β). Determination of cytokines in the supernatants was performed using ELISA. Data, expressed as percentage of control, are given as mean ± S.E.M., n = 3 for TNF-α and IL-6, and n = 5 for IL-1β. Statistics: Repeated measures ANOVA + Dunnett post hoc test with raw data. # *p* < 0.05; **,## *p* < 0.01 vs. vehicle.

For analysis of the effects of the remedies on pro-inflammatory cytokine release [19], primary monocytes isolated from human peripheral blood were pre-incubated with the test items for 30 min and then stimulated with LPS (10 ng/mL) for 4 h to measure TNF-α or for 18 h to measure IL-1β and IL-6 release in the cell supernatant by ELISA; dexamethasone (100 nM) was used as reference drug. None of the frankincense-based remedies significantly blocked the release of IL-6 or TNF-α, while dexamethasone suppressed both responses, especially IL-6 release (Figure 5). Of interest, “BOSWELLIASAN^®^” concentration-dependently inhibited the secretion of IL-1β with 55% suppression at 50 µg/mL; “Sallaki^®^ Tablets” and “H15 Ayurmedica^®^” were not significantly active at the highest test concentration of 50 µg/mL, and dexamethasone (100 nM) reduced IL-1β release by 90% (Figure 5).

## 3. Discussion

Here, we studied three frequently used frankincense-based remedies for their BA content and in parallel for inhibition of the key targets of BAs, namely 5-LOX and mPGES-1, as well as for suppression of pro-inflammatory LT and cytokine production in human primary leukocytes. We found considerable differences in the quality between the analyzed products, in terms of BA content as well as regarding their pharmacological efficiency. To our surprise “H15 Ayurmedica^®^” revealed only trace amounts of the characteristic BAs, that is, KBA, AKBA, αBA, AαBA, βBA, and AβBA, and accordingly, the pharmacological activities in vitro were much less efficient as compared to “BOSWELLIASAN^®^” and “Sallaki^®^ Tablets”. In fact, these two products contain substantial amounts of BAs and display potent pharmacological effects in vitro that might be beneficial for treatment of inflammatory diseases. Thus, the observed dual inhibition of 5-LOX and mPGES-1 along with a shift of the LM class switch towards 12/15-LOX products by the extracts is of pivotal interest with high therapeutic potential, representing a pharmacological concept which is currently pursued as a novel and innovative approach in the therapy of inflammatory diseases [20,21,22]. 

In general, ingredients of dietary supplements are presented in the same order as the corresponding amount in the product. Since the amount of *Boswellia serrata* extract per capsule is not declared on the label and based on the fact that *Boswellia serrata* is listed as penultimate ingredient prior to titanium dioxide used as colorant for the capsule shell, substantial amounts of *Boswellia serrata* extract were not expected in this product. Nevertheless, the fact that only traces of BAs could be detected fell far short of expectations. This might be attributed to inadequate extract preparation or to the application of another *Boswellia* species such as *Boswellia frereana* that contains only traces of BAs and other triterpene acids like lupeolic acid [13]. 

There is also a striking difference between the ratios of the individual BAs in the tested products. Hence, *Boswellia serrata* extracts contained in “Sallaki^®^ Tablets” and “BOSWELLIASAN^®^” in contrast to “H15 Ayurmedica^®^” are characterized by a higher amount of non-acetylated/non-ketylated BAs compared to the acetylated ones. This is clearly reflected in the ratios of the respective BAs. Thus, in both “Sallaki^®^ Tablets” and “BOSWELLIASAN^®^” the ratios of αBA/AαBA and βBA/AβBA were comparable, being 2.66 and 2.11 for αBA/AαBA in “Sallaki^®^ Tablets” and “BOSWELLIASAN^®^”, respectively, as well as 2.05 and 1.7 for βBA/AβBA “Sallaki^®^ Tablets” and “BOSWELLIASAN^®^”, respectively. In addition, the total sum of the determined BAs make up around 31% of the applied *Boswellia serrata* extract in both products. This corresponds to the known distribution and amounts of BAs in *Boswellia serrata* extracts [12,23]. On the other hand, this distribution in the individual BAs could not be detected in “H15 Ayurmedica^®^” raising further questions with regard to the origin of the extract applied in “H15 Ayurmedica^®^”.

Last but not least, it should be noticed that the determined total sum of the analyzed BAs may correspond to the declared amount of 400 mg *Boswellia serrata* for “Sallaki^®^ Tablets” and “H15 Ayurmedica^®^” or 300 mg *Boswellia serrata* extract for “BOSWELLIASAN^®^” on the label. Note that for technical reasons, we analyzed 300 mg of the *Boswellia serrata* extract contained in the product “BOSWELLIASAN^®^” that is actually composed of 400 mg of this extract. Finally, different analytical methods may result in different BA contents depending on whether HPLC, a titration method, or a very specific LC-MS/MS method was applied as in the present case [24,25,26,27]. 

BAs display a multitude of pharmacological activities [4]. 5-LOX and mPGES-1 are well-defined molecular targets that directly bind BAs and are inhibited by these triterpene acids [4,18,28,29]. mPGES-1 is an inducible enzyme in the biosynthesis of pro-inflammatory PGE_2_ from COX-2-derived PGH_2_, and is proposed as an alternative drug target to COX enzymes that are blocked by NSAIDs, to effectively and safely intervene with inflammatory disorders [30,31]. In fact, lipophilic extracts of various *Boswellia* species blocked mPGES-1 activity [32] and the abundant β-BA efficiently inhibited PGE_2_ under inflammatory conditions in vivo after oral application to rats [29]. Recently, we showed that AKBA binds an allosteric site in 5-LOX thereby shifting the regiospecificity towards a 12-lipoxygenating enzyme, which in neutrophils led to decreased LT and 5-HETE levels but increased the amounts of 12/15-LOX products and elevated anti-inflammatory specialized pro-resolving mediators (SPM) in 5-LOX-transfected HEK cells [18]. Such a pattern was also revealed in the present study for “Sallaki^®^ Tablets” and “BOSWELLIASAN^®^” that inhibited the formation of LTs and 5-HETE in human primary neutrophils that had been challenged by physiological relevant bacterial exotoxins [16] and at the same time, shifted AA conversion towards 12-HETE, as observed for AKBA [18]. BAs were shown to inhibit constitutively activated NF-kappaB signaling by blocking IkappaB kinase activity [33], which might underly the suppressive effects on pro-inflammatory cytokine release [19]. Note that the suppression of cytokine release from LPS-stimulated monocytes was much less sensitive to the frankincense products as compared to LT formation, implying a subordinated effect as compared to interference with LM formation. Future analysis on these products may also reveal additional anti-inflammatory effects such as the observed interference with the formation of reactive oxygen species [34,35]. 

It is tempting to speculate that “Sallaki^®^ Tablets” and “BOSWELLIASAN^®^” may act as LM class switch inducer also in inflammation models in vivo, that is, to lower LT and PGE_2_ levels but increase the amounts of SPM. There is a current trend towards concepts of immunoresolvent therapies in inflammation in order to support resolution [36], especially by exploiting SPM rather than blocking the inflammatory process using glucocorticoids or NSAIDs that act as immunosuppressants with severe side effects [21,22]. Our findings support the application of high-quality frankincense-based remedies in inflammatory diseases to effectively push the LM class switch towards SPM in order to promote endogenous programs of inflammation resolution without immunosuppression. Future studies with appropriate experimental models and test systems will reveal if frankincense-based remedies may act in this respect and foster SPM formation.

Finally, we point out that the strong promises of frankincense-based products in the therapy of many diseases are mainly built upon experience-based medicine, such as case reports, rather than on evidence-based medicine. Thus, there are limitations with respect to efficacy in disease treatment, for example in oncology, where the therapeutic potential for cancer treatment is still speculative and well-designed clinical studies are required to validate the clinical usefulness [6,7]. 

## 4. Materials and Methods

### 4.1. Materials

“Sallaki^®^ Tablets” (charge AB18024), “H15 Ayurmedica^®^” (charge 171), and “BOSWELLIASAN^®^” (charge HBPC11) are commercially available by various suppliers. KBA, AKBA, αBA, AαBA, βBA, and AβBA (purity > 99%) were purchased from Phytoplan (Heidelberg, Germany). Methanol of LC/MS quality was purchased from Carl Roth GmbH (Karlsruhe, Germany) and ammonium formate from Sigma Aldrich (Karlsruhe, Germany). The applied water was purified by using a Milli-Q-purification system from Merck Millipore Corporation (Bedford, MA, USA). All other chemicals and reagents were obtained from Sigma-Aldrich (Munich, Germany), unless stated otherwise. 

### 4.2. Standard Preparation

Stock standard solutions of each BA were prepared by weighing into a 20 mL volumetric flask 20 mg of each BA standard and diluting it with 20 mL methanol to yield a concentration of 1 mg/mL of each BA. Mixed spike solutions were prepared by mixing the appropriate amount of each BA stock standard solution with methanol to yield spike solutions K1 (4 µg/mL), K2 (12 µg/mL), and K3 (24 µg/mL).

### 4.3. Sample Preparation

For “Sallaki^®^ Tablets” and “BOSWELLIASAN^®^”, the contents of the tablets/capsules were pulverized and well mixed. An equivalent to 100 mg extract was weighed in a 50 mL centrifuge tube (Eco, PP, Roth, Art. AN78.1). In the case of the “H15 Ayurmedica^®^” which is an oily substance, 1 capsule was carefully cut in half in a 50 mL centrifuge glass with a scalpel. Then, 20 mL of methanol (volumetric pipette) and two glass pearls were added to each product and shaken for 60 min at 200 rpm on a vertical shaker. Afterwards, the samples were treated in an ultrasonic bath for 30 min and centrifuged for 10 min at 2000 rpm. Four aliquots of 100 µL (or 25 µL depending on the content in the oil capsules) of the clear supernatant were then transferred into four 10 mL volumetric flasks. Three of these aliquots for each product were diluted with 1 mL of the three spike solutions K1, K2, K3, respectively, to yield three differently spiked samples. To one aliquot, no spike solution was added. Finally, 20 µL of each sample solution was injected into the chromatographic system.

### 4.4. LC-MS/MS Method

The BA content was determined according to a previously developed sensitive LC-MS/MS method. In brief, LC was performed on an Agilent 1200 series consisting of a gradient pump with vacuum degasser, an autosampler, and a column oven. A Hypersil BDS RP C18 column (100 × 4 mm; 3 µm; Thermo scientific) and an upstream Gemini security guard cartridge (4 × 3 mm; Phenomenex, Germany) were used for chromatography. Separation was achieved using a gradient program starting with 90% mobile phase A (methanol: water 90:10, 400 mg/L ammonium formate) and 10% mobile phase B (methanol: water 80:20, 400 mg/L ammonium formate) rising to 100% mobile phase A within 20 min. This was kept constant for 14 min before returning to the initial conditions during 1 min. The total run time was 35 min at a flow rate of 0.4 mL/min. The column oven was set to 40 °C and the autosampler was kept at room temperature.

MS analysis was performed in the negative single ion mode (SIM, AKBA *m*/*z* 511.5, AαBA and AβBA *m*/*z* 497.4, KBA *m*/*z* 469.3, αBA and βBA *m*/*z* 455.5) on an Agilent Triple Quadrupole LC/MS 6410 series (Agilent Technologies, Waldbronn, Germany) equipped with an Electro Spray Ionization source (ESI). Dwell time was chosen to be 200 ms. The Mass Hunter software was used for data acquisition and processing. 

A standard addition method at three concentration levels (4 µg/mL, 12 µg/mL, and 24 µg/mL) was chosen for the quantification of BAs. The resulting plots of concentration versus peak area were linear with R^2^ > 0.9984 for all BAs. The percent recovery, determined by comparing the calculated amounts of the BAs with the real amount spiked to the samples, ranged from 96.87% to 100.03% for KBA, 99.94% to 100.11% for AKBA, 99.89% to 100.56% for βBA, 98.43% to 100.87% for αBA, 99.98% to 100.01% for AβBA, and 97.70% to 101.28% for AαBA. The reproducibility in terms of relative standard deviation at each level ranged from 0.24% to 2.12% for KBA, 0.2% to 1.83% for AKBA, 0.48% to 4.39% for βBA, 0.3% to 2.72% for αBA, 0.29% to 2.64% for AβBA, and 0.27% to 2.46% for AαBA.

### 4.5. Expression, Purification, and Activity Assay of Human Recombinant 5-LOX

Human recombinant 5-LOX was expressed in *E. coli* BL21 (DE3) transformed with pT3-5LO plasmid, and purification of 5-LOX was performed as described before [37]. Briefly, *E. coli* were lysed in 50 mM triethanolamine/HCl pH 8.0 plus EDTA (5 mM), soybean trypsin inhibitor (60 µg/mL), phenylmethanesulphonyl fluoride (1 mM), dithiothreitol (1 mM), and lysozyme (1 mg/mL) and then sonicated (3 × 15 s). The homogenate was then centrifuged at 40,000× *g* for 20 min at 4 °C. 5-LOX in the supernatant was partially purified by affinity chromatography on an ATP-agarose column (Sigma Aldrich, Munich, Germany). Semi-purified 5-LOX was diluted in PBS containing EDTA (1 mM) and immediately used for activity assays. 

5-LOX (0.5 µg/mL) was pre-incubated with the test items for 15 min at 4 °C. 5-LOX product formation was initiated by addition of 2 mM CaCl_2_ plus 20 µM arachidonic acid. After 10 min at 37 °C, the reaction was terminated by adding 1 mL ice-cold methanol. Formed 5-LOX metabolites (all-trans isomers of LTB_4_ and 5-hydro(peroxy)eicosatetraenoic acid (H(P)ETE)) were analyzed by RP-HPLC as described [38].

### 4.6. Induction of mPGES-1 in A549 Cells, Isolation of Microsomes, and Determination of mPGES-1 Activity

Microsomes of IL-1β-activated A549 cells were prepared and mPGES-1 activity determined as described [29]. In brief, cells were treated with IL-1β (1 ng/mL) at 37 °C and 5% CO_2_, harvested after 72 h, and frozen in liquid nitrogen. After reuptake of the cells in ice-cold homogenization buffer (0.1 M potassium phosphate buffer pH 7.4, 1 mM phenylmethanesulphonyl fluoride, 60 µg/mL soybean trypsin inhibitor, 1 µg/mL leupeptin, 2.5 mM glutathione, and 250 mM sucrose) and incubation for 15 min, cells were sonicated on ice (3 × 20 s) and subjected to differential centrifugation at 10,000× *g* for 10 min and 174,000× *g* for 1 h at 4 °C. The microsomal fraction (pellet) was resuspended in homogenization buffer, analyzed for its protein content using a protein assay kit (Bio-Rad laboratories GmbH, Munich, Germany), and diluted in potassium phosphate buffer (0.1 M, pH 7.4) containing 2.5 mM glutathione. After pre-incubation with the test items for 15 min at 4 °C, the reaction (100 µL total volume) was initiated by the addition of 20 µM PGH_2_ and terminated after 1 min by the addition of 100 µL stop solution (40 mM FeCl_2_, 80 mM citric acid, and 10 µM of 11β-PGE_2_ as internal standard). PGE_2_ was separated by solid phase extraction on RP-C18 material using acetonitrile (200 µL) as eluent, and analyzed by RP-HPLC (30% acetonitrile aqueous, 0.007% TFA (*v*/*v*), Nova-Pak^®^ C18 column, 5 × 100 mm, 4 µm particle size, flowrate 1 mL/min) with UV detection at 195 nm.

### 4.7. Isolation of Human Leucocytes

Leucocyte concentrates from freshly withdrawn peripheral blood of adult healthy donors were provided by the Institute of Transfusion Medicine of the University Hospital Jena, Germany. The experimental protocol was approved by the ethical committee of the University Hospital Jena. All methods were performed in accordance with the relevant guidelines and regulations. Neutrophils and monocytes were immediately isolated as described before [39]. In brief, cells were isolated by dextran sedimentation and Ficoll-Histopaque 1077-1 (Sigma-Aldrich) density centrifugation. To purify neutrophils, the remaining erythrocytes were removed by hypotonic lysis. Neutrophils were finally resuspended in PBS pH 7.4 plus 1 mg/mL glucose at the cell density of 5 × 10^6^ cells /mL. Monocytes were separated from peripheral blood mononuclear cells by adherence to cell culture flasks (Greiner Bio-one, Frickenhausen, Germany) for 1 h (37 °C, 5% CO_2_), followed by cell scraping and resuspension in RPMI 1640 supplemented with 5% fetal calf serum, 2 mmol/L L-glutamine (Biochrom/Merck, Berlin, Germany), and 100 U/mL penicillin; 100 µg/mL streptomycin (Biochrom/Merck).

### 4.8. Preparation of Exotoxin-Containing Staphylococcus Aureus-Conditioned Medium (SACM)

A single colony of *Staphylococcus aureus* (strain 6850) grown for 24 h at 37 °C on Columbia-agar plates containing 5% sheep blood (Altmann Analytic, Munich, Germany) was picked and inoculated in 50 mL of brain heart infusion broth (BHI, Sigma-Aldrich) for a further 24 h at 37 °C under shaking (250 rpm). The OD_600_ was adjusted to 0.05 by dilution with BHI and 50 mL of the corresponding solution was cultivated for another 24 h. Finally, 15 mL were centrifuged for 10 min at 3350× *g* and sterile filtered using a 0.22 µm PVDF syringe filter (Carl Roth GmbH).

### 4.9. Analysis of LOX Product Formation in Human Neutrophils

Human neutrophils (5 × 10^6^/mL) were preincubated with a test item or vehicle (0.1% EtOH) for 15 min at 37 °C. The production of LOX products was induced by addition of SACM (1%) and CaCl_2_ (1 mM). After 90 min at 37 °C, the reaction was stopped with 2 mL ice-cold MeOH containing deuterium-labeled internal standards d_8_-5S-HETE and d_4_-LTB_4_ (200 nM each), and 10 µM d_8_-AA (Cayman Chemical/Biomol GmbH, Hamburg, Germany). Sample preparation was conducted as described previously [40]. In brief, samples were kept at −20 °C overnight to allow protein precipitation. After centrifugation (1200× *g*, 4 °C, 10 min), supernatants were transferred to 7 mL acidified H_2_O (pH 3.5, HCl). The LOX products in these samples were purified by solid phase extraction (SPE). Solid-phase C18 cartridges (Sep-Pak^®^ Vac 6cc 500 mg/ 6 mL C18; Waters, Milford, MA) were equilibrated with 6 mL MeOH and 6 mL H_2_O. Next, samples were loaded onto the columns. After washing with 6 mL H_2_O and additional 6 mL *n*-hexane, LMs were eluted with 6 mL methyl formate. Finally, the samples were brought to dryness using an evaporation system (TurboVap LV; Biotage, Uppsala, Sweden) and resuspended in 100 µL methanol-water (50/50, *v*/*v*) for ultraperformance liquid chromatography–tandem mass spectrometry measurements (UPLC–MS-MS). LOX product profiling was conducted by means of an Acquity™ UPLC system (Waters, Milford, MA, USA) and a QTRAP 5500 Mass Spectrometer (ABSciex, Darmstadt, Germany) equipped with a Turbo V™ Source and electrospray ionization. LM were eluted using an ACQUITY UPLC^®^ BEH C18 column (1.7 µm, 2.1 × 100 mm; Waters, Eschborn, Germany) at 50 °C with a flow rate of 0.3 mL/min and a mobile phase consisting of methanol-water-acetic acid of 42:58:0.01 (*v*/*v*/*v*) that was ramped to 86:14:0.01 (*v*/*v*/*v*) over 12.5 min and then to 98:2:0.01 (*v*/*v*/*v*) for 3 min. The QTrap 5500 was operated in negative ionization mode using scheduled multiple reaction monitoring (MRM) coupled with information-dependent acquisition. Optimized LM parameters (CE, collision energy; EP, entrance potential; DP, declustering potential; CXP, collision cell exit potential) were adopted [40], and the curtain gas pressure was set to 35 psi. The retention time and diagnostic ions for each LM were confirmed by means of an external standard (Cayman Chemical/Biomol GmbH, Hamburg, Germany). Quantification was achieved by calibration curves for each LM.

### 4.10. Determination of Cytokine Levels

Monocytes (1.5 × 10^6^/mL) were resuspended in monocyte medium (RPMI 1640 supplemented with 5% fetal calf serum, 2 mmol/L L-glutamine (Biochrom/Merck, Berlin, Germany), 100 U/mL penicillin, and 100 µg/mL streptomycin (Biochrom/Merck)). After 1.5 h at 37 °C and 5% CO_2_, test items or vehicle were added for 30 min. Then, cells were stimulated with 10 ng/mL LPS for 4 h (TNF-α) or 18 h (IL-1β and IL-6). For measurement of extracellular cytokine levels, the supernatants were collected and centrifuged (2000× *g*, 4 °C, 10 min). Cytokines in the supernatant were analyzed by in-house-made ELISA using capture and detection antibodies from R&D Systems (Abington, UK).

## Figures and Tables

**Table 1:** Content of the six characteristic boswellic acids in frankincense products quantified by LC-MS/MS. The amounts of declared extract and of BAs are given in mg/tablet or capsule, each.

Name	Batch	Dosage Form	Declared Extract Content	KBA	AKBA	αBA	β-BA	AαBA	AβBA	∑ of BA
Sallaki^®^ tablets	AB18024	tablets	400	9.34	7.88	20.73	52.65	7.77	25.72	124.07
H15 Ayurmedica^®^	171	oily capsules	400	0.30	0.31	0.40	0.27	0.33	0.32	1.92
BOSWELLIASAN^®^	HBPC11	hard gelatin capsule	300	5.12	7.51	13.58	36.40	6.45	21.16	90.21

## Data Availability

The data presented in this study are available on reasonable request from the corresponding author. The data are not publicly available due to privacy.
